# Can We Finally Identify the Psychological Effect of Body Size? A Century From Kretschmer to Mendelian Randomization

**DOI:** 10.2188/jea.JE20250679

**Published:** 2026-10-05

**Authors:** Zui C Narita, Tomohiro Shinozaki, Toshi A Furukawa, Atsushi Nishida

**Affiliations:** 1 Department of Behavioral Medicine, National Institute of Mental Health, National Center of Neurology and Psychiatry, Tokyo, Japan; 2 Interfaculty Initiative in Information Studies and Department of Biostatistics, School of Public Health, Graduate School of Medicine, The University of Tokyo, Tokyo, Japan; 3 Department of Health Promotion and Human Behavior, Kyoto University Graduate School of Medicine and School of Public Health, Kyoto, Japan; 4 Unit for Mental Health Promotion, Research Center for Social Science & Medicine, Tokyo Metropolitan Institute of Medical Science, Tokyo, Japan

**Keywords:** obesity, overweight, thinness, mental health, randomized controlled trials

The psychological effect of body size has long been a topic of scientific interest. From Kretschmer’s *Physique and Character*^1^ to contemporary analyses, researchers have been pursuing this question for over a century. In this article, we critically reflect on whether contemporary approaches genuinely reveal the psychological effect of body size and suggest alternative directions that may be more fruitful for future scientific inquiry.

What exactly is the psychological effect? It is the counterfactual contrast between what would happen psychologically if the exposure were present versus if it were not. While we cannot simultaneously observe both outcomes in the same individual or group, a randomized controlled trial (RCT) allows us to observe outcomes in exchangeable groups. Naturally, the definition of an intervention needs to be sufficiently precise,^2^ and the intervention must be manipulable in terms of whether it occurs or not.^3^ Imagine a hypothetical scenario where we can conduct an ideal RCT with treatment assignments: attaining a body mass index (BMI) ≥25 kg/m^2^ versus maintaining a BMI <25 kg/m^2^. Here, participants have perfect adherence to treatment assignments, and the two treatment groups follow the same protocol except for BMI control. Even under such ideal conditions, we still cannot measure the psychological effect of being overweight because the definition of the intervention is not sufficiently precise; a BMI ≥25 kg/m^2^ could result from various interventions, such as dietary behavior or muscle training. Each of these interventions would have a different psychological effect; the psychological effects are not identifiable unless all versions of the interventions are measured within the data.

Mendelian randomization (MR) has provided new approaches to investigate the association between body size and mental health. MR is often justified by analogy to RCTs, under the premise that genetic factors are randomly assigned by nature. In this framework, the psychological effects of body size are considered analyzable by virtue of “randomly assigned” genetic variants. Nevertheless, it cannot provide answers to research questions that fall outside the scope of what an actual RCT could investigate. In fact, MR may introduce additional limitations, such as confounding bias, selection bias, and violation of the exclusion restriction assumption.^4^^–^^7^ MR would virtually never be as robust as an RCT, even if it could ever function as its analogue at best. Recognizing this helps demystify MR, which is sometimes mistakenly treated as capable of addressing research questions that RCTs themselves could not.

While it is infeasible to measure the psychological effect of body size via RCTs or MR, mediation analysis^8^ and interaction analysis^9^ offer alternative frameworks that may yield more useful insights. In these frameworks, body size is not the exposure of interest; well-defined interventions, such as exercise programs, are. Body size is analyzed as (1) a mediator through which an intervention may influence psychological outcomes or (2) an effect modifier of the intervention’s psychological effects. Mediation analysis enables the decomposition of the total effect of an intervention into a direct effect and an indirect effect through body size, thereby reflecting the importance of body size-related mechanisms. For instance, consider a setting in which exercise programs are the exposure, depression is the outcome, and body size is a potential mediator; researchers can then examine the proportion of the total effect of exercise programs on depression that is mediated by body size. In principle, causal mediation analysis, including controlled, natural, and interventional direct and indirect effects, relies on a well-defined intervention on the mediator to define the effects based on counterfactual outcomes in which the mediator is set to specific values. Nevertheless, natural and interventional effects would offer the decomposed portions through the mediator values that arise endogenously in the study population under the specific value of the exposure. Separable effects^2^ may further circumvent the requirement of a well-defined intervention on the mediator by *hypothetically* separating the exposure into two components: (1) one with an effect on the outcome but no effect on the mediator, and (2) the other with an effect on the mediator but no direct effect on the outcome. The separable direct and indirect effects can be identified as long as well-defined interventions on these two components are available at least hypothetically. Interaction analysis can address a different but complementary question: whether the psychological effect of an intervention differs across levels of baseline body size. For instance, consider a setting in which exercise programs are the exposure, depression is the outcome, and baseline body size is a potential effect modifier; researchers can then examine which subgroup of body size experiences the largest effect of exercise programs on depression. The effect modification on an additive scale is commonly assessed by computing an estimate referred to as relative excess risk due to interaction. Note that in this approach, we can address effect modification rather than an interaction for joint intervention on exposure and body size. Both mediation and interaction analyses can be applied not only to RCTs but also to observational studies; their application in the latter requires particular caution regarding the definition of well-defined intervention on exposure, as well as confounding bias, selection bias, and immortal time bias due to misalignment of timings of eligibility assessment, exposure classification, and the start of follow-up (ie, “time zero”).

From Kretschmer’s era to the present day, a century of scientific advancement has perhaps taught us why and how the psychological effect of body size is unidentifiable. Rather than pursuing the measurement of an effect that is fundamentally unidentifiable even via contemporary approaches, researchers and scientific communities may consider shifting their focus toward more practically useful inquiries, which are addressable via alternative contemporary approaches.
